# Cellular response of chondrocytes to magnesium alloys for orthopedic applications

**DOI:** 10.3892/ijmm.2015.2211

**Published:** 2015-05-14

**Authors:** YI LIAO, QINGLI XU, JIAN ZHANG, JIALING NIU, GUANGYIN YUAN, YAO JIANG, YAOHUA HE, XINLING WANG

**Affiliations:** 1 Department of Orthopaedics, The Fifth People’s Hospital of Shanghai, Fudan University, Shanghai 200240, P.R. China; 2 Department of Orthopaedics, The Huashan Hospital Baoshan Branch, Fudan University, Shanghai 200431, P.R. China; 3 National Engineering Research Center of Light Alloys Net Forming (LAF), School of Materials Science and Engineering, Shanghai Jiao Tong University, Shanghai 200240, P.R. China; 4 Department of Orthopaedics, The Sixth People’s Hospital, Shanghai Jiao Tong University, Shanghai 200233, P.R. China; 5 Department of Radiology, The Fifth People’s Hospital of Shanghai, Fudan University, Shanghai 200240, P.R. China

**Keywords:** Mg-Nd-Zn-Zr alloy, magnesium alloy, chondrocytes, cytotoxicity, *in vitro*

## Abstract

In the present study, the effects of Mg-Nd-Zn-Zr (JDBM), brushite (CaHPO_4_·_2_H_2_O)-coated JDBM (C-JDBM), AZ31, WE43, pure magnesium (Mg) and Ti alloy (TC4) on rabbit chondrocytes were investigated *in vitro*. Adhesion experiments revealed the satisfactory morphology of chondrocytes on the surface of all samples. An indirect cytotoxicity test using MTT assay revealed that C-JDBM and TC4 exhibited results similar to those of the negative control, better than those obtained with JDBM, AZ31, WE43 and pure Mg (p<0.05). There were no statistically significant differences observed between the JDBM, AZ31, WE43 and pure Mg group (p>0.05). The results of indirect cell cytotoxicity and proliferation assays, as well as those of apoptosis assay, glycosaminoglycan (GAG) quantification, assessment of collagen II (Col II) levels and RT-qPCR revealed a similar a trend as was observed with MTT assay. These findings suggested that the JDBM alloy was highly biocompatible with chondrocytes *in vitro*, yielding results similar to those of AZ31, WE43 and pure Mg. Furthermore, CaHPO_4_·_2_H_2_O coating significantly improved the biocompatibility of this alloy.

## Introduction

Magnesium (Mg) and its alloys are susceptible to dissolution in aqueous solutions due to their extremely low corrosion potential, particularly in alloys containing chloride ion electrolytes (1). For this reason, Mg alloys have attracted considerable attention as potential implant materials (2–8). The interest in Mg alloys has been primarily motivated by their biocompatibility, biodegradability and desirable mechanical properties. For instance, the tensile strength and elastic modulus of Mg alloys are closer to those of bone, as compared with the commonly used steel and titanium alloys (2–8). A previous *in vivo* study suggested that Mg alloy implantation in animal models promoted new bone formation and biocompatibility (2). Therefore, Mg and Mg alloys may be used as biodegradable materials for orthopedic implants.

The Mg alloys that have been investigated as implant materials are mostly commercial alloys designed for the transportation industry (8). Most of these commercial Mg alloys contain aluminium (Al) and rare earth metals. However, Al is neurotoxic (9), whereas severe hepatotoxicity has been detected following the administration of rare earth metals (10). Therefore, the exploration of novel non-toxic or low-toxicity Mg alloy systems has become a research highlight.

In this study, a medical Mg alloy, designated as Mg-Nd-Zn-Zr (JDBM), was further evaluated for its cytocompatibility *in vitro.* This alloy contains a Mg matrix and approximately 3% rare earth metals (11). Preliminary results revealed that JDBM possessed favorable *in vitro* biocompatibility to rabbit chondrocytes (12). In this study, rabbit chondrocytes were further used as an *in vitro* model to evaluate the effects of JDBM, brushite (CaHPO_4_·_2_H_2_O)-coated JDBM (C-JDBM), AZ31, WE43, pure Mg and Ti alloys (TC4). The effects of the Mg and Mg alloys on the adhesion, viability, proliferation and apoptosis of chondrocytes were investigated. The glycosaminoglycan (GAG) and collagen II (Col II) content, as well as the mRNA expression of Col II and aggrecan were also investigated.

## Materials and methods

### Sample and extract preparation

JDBM was previously developed for biomaterial applications by our group (11). C-JDBM, the commercial Mg alloys, WE43 and AZ31, pure Mg (Shanxi Yanbixin Magnesium Co., Ltd., Shanxi, China), and the Ti alloy, TC4 (Daiyuan, Shanghai, China), were analyzed for comparison. The detailed preparation of these materials has been described in our previous study (11).

Disk samples, 10 mm in diameter and 2.0 mm in height, were obtained by electrode discharge machining from the extruded JDBM, WE43, AZ31 and TC4 rods, as well as from high-purity Mg ingots (99.99%). All samples were ground using SiC paper of up to 1,200 grit and polished with 1 *µ*m diamond abrasive paste, followed by a series of ultrasonic cleaning in acetone, ethanol and distilled water. The details of the CaHPO_4_·_2_H_2_O treatment of the JDBM disc, including the fabrication of the coating and the evaluation of its properties are described in another study of ours (13). Prior to the *in vitro* cytocompatibility experiments, the samples were sterilized with ethylene oxide for 24 h.

Sample extracts were prepared according to ISO 10993. The disk samples were immersed in 1.7584 ml Dulbecco’s modified Eagle’s medium with F12 (DMEM/F12) supplemented with 10% fetal bovine serum (FBS) (both from Gibco-BRL, Carlsbad, CA, USA), with a surface area-to-extraction medium volume ratio of 1.25 cm^2^/ml. The samples were then incubated in a humidified atmosphere with 95% humidity and 5% CO_2_ at 37°C for 72 h. The supernatant was collected, and the obtained extracts were refrigerated at 4°C and used within 3 days.

### Chondrocyte harvest and culture

Chondrocytes were isolated from aseptically harvested articular cartilage from the knee joints of adult New Zealand rabbits weighing 2.0–2.5 kg. The cartilage samples were rinsed in sterile phosphate-buffered saline (PBS) (pH 7.4; Gibco-BRL) containing penicillin and streptomycin (100 U/ml and 100 *µ*g/ml, respectively) (HuaBei, Shijiazhuang, China). The samples were then cut into 1–2 mm^3^ fragments, placed in a spinner flask containing 0.25% trypsin-EDTA (Gibco-BRL) at 37°C for 30 min, and rinsed thrice in sterile PBS. The slices as prepared samples were subsequently digested with 0.2% Col II (Sigma, St. Louis, MO, USA) in sterile PBS at 37°C for a further 12–16 h. The chondrocytes were then harvested, counted and seeded onto 25 cm^2^ culture flasks at a cell density of 2×10^4^/cm^2^ in DMEM/F12 with 10% FBS (Gibco-BRL). The cells were then cultured in an incubator (Thermo 8000; Thermo Fisher Scientific, Inc., Shanghai, China) at 37°C with 95% humidity and 5% CO_2_ for 7–10 days. The cell culture medium was replenished every 3 days. The cells were passaged when they reached 80–90% confluence. Cells at passage 2 were used for further experiments.

### Cell adhesion assay

Cell suspensions of 1.5 ml each were seeded into 24-well plates containing the JDBM, C-JDBM, AZ31, WE43, pure Mg and TC4 disc samples, at a cell density of 1×10^5^ cells/ml. The culture medium was replenished daily. The cultures were incubated in a humidified atmosphere (95%) with 5% CO_2_ at 37°C. The samples were collected after 1 and 3 days of incubation and washed thrice with PBS (pH 7.4; Gibco-BRL) to remove the non-adherent cells. The cells were then fixed in 2.5% glutaraldehyde (Boster, Wuhan, China) solution at room temperature for 2 h followed by rinsing thrice with PBS. Following gradient ethanol dehydration (50, 60, 70, 80, 90 and 100%; for 10 min gradient ethanol dehydration), the samples were dried in a hexamethyldisilazane solution (Wuhan Boster Biological Technology, Ltd.). The samples were subsequently sputter-coated with gold. The surfaces of the cell-adhered experimental samples were observed by scanning electron microscopy (SEM; FEI Quanta 250; FEI Co., Hillsboro, OR, USA). Three parallel samples were used for each experimental condition.

Another set of 6 parallel samples were treated according to the method described above. However, these samples were fixed in 4% paraformaldehyde solution (Wuhan Boster Biological Technology, Ltd.) at room temperature for 30 min and rinsed thrice with PBS (pH 7.4; Gibco-BRL), followed by staining with a 4′,6-diamidino-2-phenylindole (DAPI) solution (5 *µ*g/ml) (Beyotime Institute of Biotechnology, Jiangsu, China) for 10 min. The samples were again rinsed thrice with PBS, for 5 min for each wash. The surfaces of the cell-adhered experimental samples were observed and recorded using an inverted phase contrast microscope (Olympus IX70; Olympus Corp., Tokyo, Japan).

### Indirect cell cytotoxicity and proliferation assay

The cells cultured in DMEM/F12 alone were used as the negative controls, whereas those cultured in DMEM/F12 medium with 10% DMSO (Beyotime Institute of Biotechnology) were used as the positive controls. The cells were incubated in 96-well cell culture plates at 5×10^3^ cells per 100 *µ*l medium in each well for 24 h to allow attachment. The culture medium was then replaced with 100 *µ*l of the extraction medium with the respective treatments. The 96-well cell culture plates were then observed under an optical microscope (CSW-30B; Shenzhen Coosway Optical Technology Co., Ltd, Shenzhen, China) on days 1, 3 and 5 following incubation. The culture medium was replenished every 2 days. Six parallel wells were established for each tested sample. A total of 20 *µ*l 3-(4,5-dimethylthiazol-2-yl)-2,5-diphenyltetrazolium bromide (MTT) (Sigma) solution (5 mg/ml in PBS) was added to each well. The samples were then placed in a cell incubator for 4 h. Subsequently, 100 *µ*l of the formazan solubilization solution [10% SDS in 0.01 MHCl (Sigma)] were added to each well and the plates were again incubated overnight in a cell incubator with a humidified atmosphere (95%) and 5% CO_2_ at 37°C. The spectrophotometric absorbance of the samples at 570 nm was measured using a microplate reader (Bio-Rad 680; Bio-Rad, Hercules, CA, USA).

Another set of 6 parallel 96-well cell culture plates were used to observe and record the cell morphology. The test condition was the same the as one described above, and the medium was replaced with 100 *µ*l extraction medium. Following incubation for 1, 3 and 5 days, the 96-well cell culture plates were collected and rinsed thrice with PBS (pH 7.4; Gibco-BRL), and then stained with Alcian blue and DAPI solution (5 *µ*g/ml) (both from Beyotime Institute of Biotechnology). The cell morphologies of the samples in the 96-well cell culture plates were observed and recorded using an inverted phase contrast microscope (Olympus IX70; Olympus Corp.). Three parallel wells were established for each stain.

### Cell apoptosis assay

The chondrocytes were analyzed as follows: 7 treatment groups were set, namely, a negative control (DMEM/F12 medium), a JDBM, C-JDBM, AZ31, WE43 pure Mg and TC4 group. Cell suspensions (10 ml) were seeded into 75 cm^2^ culture flasks at a cell density of 1×10^5^ cells/ml and incubated for 24 h to allow attachment. The medium was then replaced with 10 ml extraction medium or DMEM/F12 medium. The culture media were replaced after 2 days with 10 ml. Three parallel culture flasks were established for each treatment condition. Following incubation of the cells in a cell incubator for 5 days, the culture flasks were observed under an optical microscope. Chondrocyte apoptosis was assessed using the Alexa Fluor^®^ 488 Annexin V/Dead Cell Apoptosis kit (Invitrogen Life Technologies, Carlsbad, CA, USA) with Alexa Fluor^®^ 488 Annexin V and propidium iodide (PI) (Beyotime Institute of Biotechnology) for flow cytometry (Navios; Beckman Coulter, Inc., Brea, CA, USA). The assay was performed according to the manufacturer’s instructions.

### GAG quantification assay

The quantification of the GAG content was conducted by a modification of the dimethylmethylene blue method (Beyotime Institute of Biotechnology) (14). The chondrocytes were treated as follows: the cells were seeded in 6-well cell culture plates at a density of 1×10^5^ cells/well and incubated for 24 h to allow attachment. The medium was changed with 3 ml of extraction medium every 2 days. Following incubation of the cells under the cell culture conditions for 5 days, the 6-well cell culture plates were observed under an optical microscope. Three parallel wells were established for each treatment condition. After rinsing in PBS (pH 7.4; Gibco-BRL), a cytolysate (RIPA; Wuhan Boster Biological Technology, Ltd.) was added to each well at 0.4 ml/well and vortexed for 10 sec. An aliquot (40 *µ*l) of the digest was assayed for the total GAG content by the addition of 200 *µ*l of 1,9-dimethylmethylene blue dye solution (Beyotime Institute of Biotechnology). The absorbance was determined at 595 nm using a microplate reader (Bio-Rad 680; Bio-Rad). The amount of GAG was extrapolated from a standard curve based on shark chondroitin sulfate.

### Col II content assay and enzyme-linked immunosorbent assay (ELISA)

The chondrocytes were treated as described above. The cells were seeded in 12-well plates at a density of 5×10^4^ cells/well. The cells were then incubated for 24 h to allow attachment. The medium was then changed with 2 ml of extraction medium every 2 days. Following incubation of the cells in a humidified atmosphere (95%) with 5% CO_2_ at 37°C for 5 days, the 12-well cell culture plates were observed under an optical microscope. Three parallel wells were established for each treatment condition. After the cell culture plates were placed on ice and rinsed in PBS (pH 7.4; Gibco-BRL), a cytolysate (RIPA; Wuhan Boster Biological Technology, Ltd.) was added to each well at 0.2 ml/well, vortexed for 10 sec, and centrifuged for 10 min at 10,000 x g according to the manufacturer’s instructions. An ELISA kit (R&D Systems, Minneapolis, MN, USA) was used for the quantitative determination of rabbit Col II levels in the cell cultures from each well. ELISA was conducted according to the manufacturer’s instructions. The spectrophotometric absorbance of the samples at 450 nm was measured using a microplate reader (Bio-Rad 680; Bio-Rad). The standard curve was calculated according to the concentration and absorbance of the standard, asw ell as based on the curve to extrapolate the corresponding concentration of the sample.

### RNA extraction and quantitative-reverse transcription PCR (RT-qPCR)

The chondrocytes were seeded into 25 cm^2^ culture flasks at a density of 4×10^5^ cells per flask and incubated for 24 h to allow attachment. The medium was then changed with 5 ml of the respective extracts every 2 days. DMEM/F12 medium was used as a negative control. Following incubation of the cells in a humidified atmosphere (95%) with 5% CO_2_ at 37°C for 5 days, the culture flasks were observed under an optical microscope. Three parallel wells were established for each treatment condition. Total RNA from the chondrocytes in the different groups was isolated using TRIzol reagent (Invitrogen Life Technologies). First-strand cDNA was synthesized using the RevertAid First Strand cDNA Synthesis kit (Thermo Fisher Scientific, Inc., Waltham, MA, USA), as previously described (12). Quantitative PCR (qPCR) was performed to amplify rabbit GAPDH, aggrecan and Col II using the LightCycler DNA Master SYBR-Green I kit (Roche, Basel, Switzerland) according to the manufacturer’s instructions. The copies of target cDNA were normalized to GAPDH expression (housekeeping gene). Each PCR reaction was repeated thrice for each independent sample. The primers used were the same as those in a previous study of ours (12).

### Statistical analysis

Three replicates were conducted for each test (cytotoxicity test, N=6) and are presented as the mean values ± standard deviation (SD). ANOVA for repeated measurements was used for the MTT assay data. Multivariate ANOVA and the multiple-comparison post hoc test [least significant difference (LSD)] were subsequently conducted for pairwise comparisons between groups at each time point. One-way ANOVA, followed by the multiple-comparison post hoc test [Student-Newman-Keul (SNK) test; Tukey’s test], was performed using SPSS (version 16.0) software for other group comparisons. Mauchly’s test of sphericity was used to determine whether there were any associations among the repeatedly measured data. If any (p≤0.05), multivariate ANOVA was then performed, or the Greenhouse-Geisser corrected results were taken into consideration. The effects of treatment were evaluated by estimating between subject variance. The repeated measurement effect or its interactive effect with the treatment group was evaluated by estimating within subject variance. The method of Bonferroni was used to perform pairwise comparisons of the repeatedly measured data at different measurement times in each treatment group. A value of p<0.05 was considered to indicate a statistically significant difference.

## Results

### Cell adhesion and morphology

**Figure 1:**
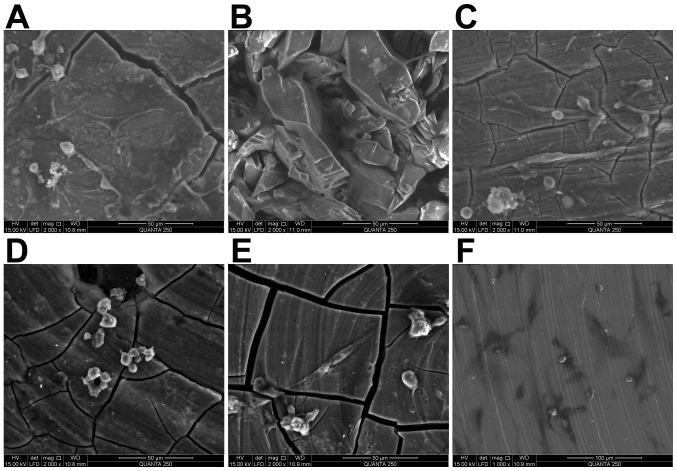
Scanning electron microscopy (SEM) of chondrocytes cultured on (A) Mg-Nd-Zn-Zr (JDBM), (B) brushite (CaHPO_4_·_2_H_2_O)-coated JDBM (C-JDBM), (C) AZ31, (D) WE43, (E) pure magnesium (Mg), and (F) TC4 alloy samples for 3 days. The chondrocytes cultured on the discs presented with a highly elongated, irregular and round shape.

**Figure 2:**
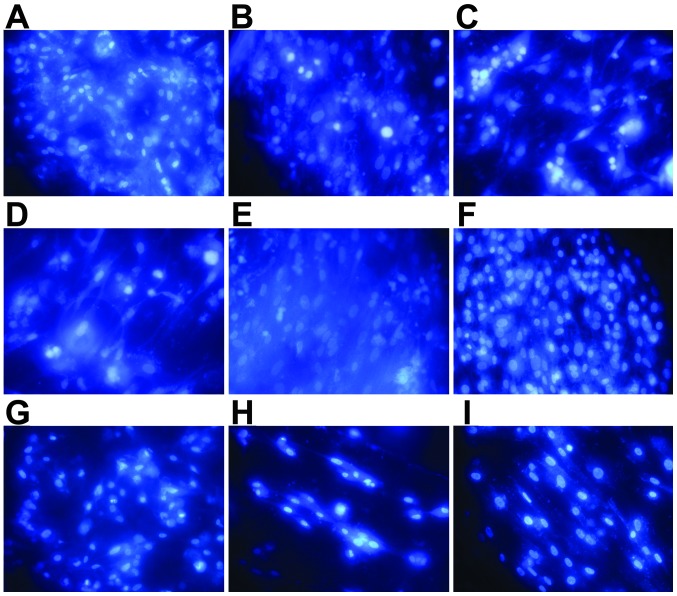
4′,6-Diamidino-2-phenylindole (DAPI) staining of chondrocytes (x400 magnification). Cells cultured for 3 days on (A) brushite (CaHPO_4_·_2_H_2_O)-co ated JDBM (C-JDBM), (B) Mg-Nd-Zn-Zr (JDBM), (C) AZ31, (D) WE43, (E) pure magnesium (Mg), and (F) TC4. Cells cultured for 1 day on (G) C-JDBM, (H) pure Mg, and (I) TC4. A few cells were already observed on the samples after 1 day of culture (G–I). After 3 days of culture, the number of cells on the discs increased (A–F). Among the cultures, a significantly greater number of adhered cells was observed on the surfaces of the C-JDBM and TC4 discs (A and F, respectively), whereas the least number of cells was observed on the WE43 disc (D).

The morphologies of the chondrocytes cultured on the discs of JDBM, C-JDBM, AZ31, WE43, pure Mg and TC4 for 1 or 3 days are illustrated in Figs. 1 and 2.

The chondrocytes cultured on the discs presented with a highly elongated, irregular and round shape. A few cells were already observed on the samples after 1 day of culture (Fig. 2G–I). After 3 days of culture, the number of cells on the discs increased (Fig. 2A–F). Among the cultures, a significantly greater number of adhered cells was observed on the surfaces of the C-JDBM and TC4 discs (Fig. 2A and F, respectively), whereas the least number of cells was observed on the WE43 disc (Fig. 2D).

### Indirect cytotoxicity and proliferation assay

**Figure 3:**
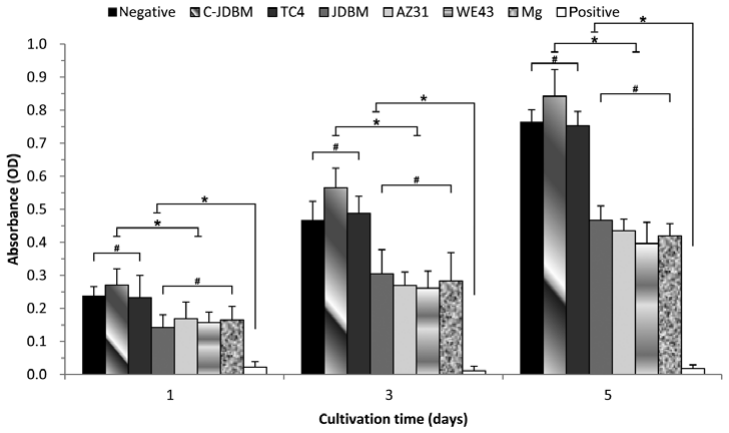
Results of cytotoxicity assay of the Mg-Nd-Zn-Zr (JDBM), brushite (CaHPO_4_·_2_H_2_O)-coated JDBM (C-JDBM), AZ31, WE43, pure magnesium (Mg), and TC4 extraction media, as wel as the Dulbecco’s modified Eagle’s medium with F12 (DMEM/F12) culture media, with and without 10% DMSO [mean ± standard deviation (SD)] on chondrocytes after 1, 3 and 5 days. ^*^P<0.05 (positive control vs. all treatments; negative control, C-JDBM, and TC4 vs. the other Mg alloys and Mg). ^#^P>0.05 (negative control vs. C-JDBM vs. TC4; JDBM vs. AZ31 vs. WE43 vs. Mg). Negative control, cells cultured in DMEM/F12 alone; positive control, cells cultured in DMEM/F12 medium with 10% DMSO.

**Figure 4:**
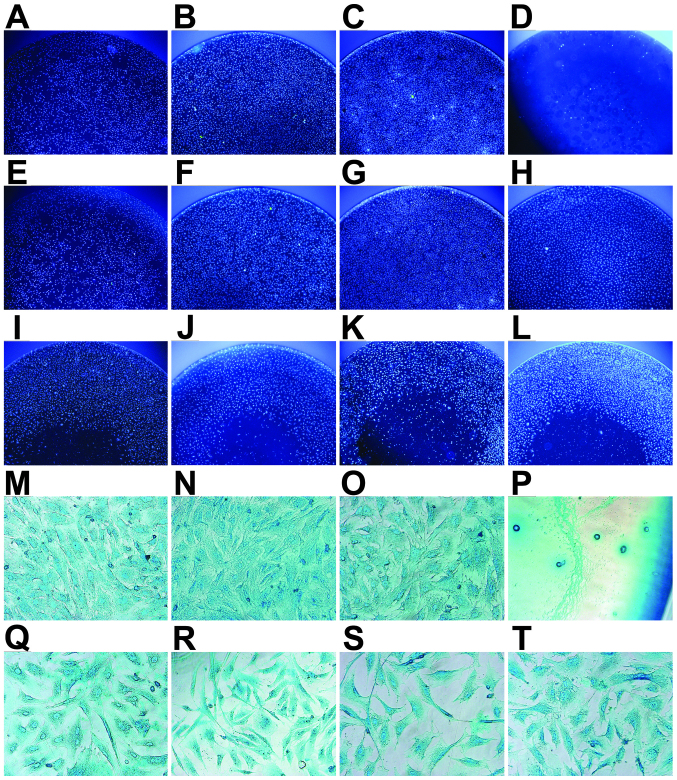
Morphology of the chondrocytes follwoing 1, 3 and 5 days of incubation with: (A–C and M) Dulbecco’s modified Eagle’s medium with F12 (DMEM/F12), (D and P) 10% DMSO medium, (E–G and N) brushite (CaHPO_4_·_2_H_2_O)-coated JDBM (C-JDBM), (H and O) Ti alloy (TC4), (I and Q) Mg-Nd-Zn-Zr (JDBM), (J and R) AZ31, (K and S) WE43, and (L and T) pure magnesium (Mg). (A–C and E–G) Cells were incubated for 1, 3 and 5 days, respectively. (D and H–T) Cells were incubated for 3 days. (A–L) Cells were stained with 4′,6-diamidino-2-phenylindole (DAPI) (x40 magnification). (M–T) Cells were stained with Alcian blue (x400 magnification). A greater number of cells was observed in the negative control group (B), C-JDBM (F) and TC4 (H) groups after 3 days, as compared with the other groups (I–L), due to the loss of cells in the center of the other culture plates, while no other differences were observed between them. (A–C and E–G) The number of cells was directly associated with the incubation time. The chondrocytes in the positive control group were extremely scarce (D and P), and unlike the elongated, polygonal, deltoid or irregular shape of the other chondrocytes (M–O and Q–T), their morphology had changed to a small and round shape (P), whereas the cells from all the other treatment groups appeared normal.

The absorbance of the chondrocytes cultured on the different extracts, DMEM/F12 and 10% DMSO media for 1, 3, and 5 days is shown in Fig. 3. The morphologies of the chondrocytes after 1, 3, and 5 days of incubation are presented in Fig. 4. As shown in Fig. 3, the absorbance of all groups of cells increased with time. As shown in Table I, the time effect (day) and the effects of day x group interaction were statistically significant (p<0.05), indicating that the research target changed with time. Furthermore, the role of the time factor varied within each group. Tests on the between-subject effects indicated that the grouping factor influenced the results, and each group of targets generally differed (p<0.05). Likewise, the absorbance of C-JDBM (Fig. 3) was greater than that of the negative control and TC4, although the difference was not significant (p>0.05). The absorbance of the remaining groups showed significantly lower results (p<0.05). By contrast, no significant differences were observed among the JDBM, AZ31, WE43 and pure Mg groups (p>0.05), thereby indicating that these alloys have high cytocompatibility with chondrocytes.

The analysis of cell morphology (Fig. 4) presented similar results as those of the MTT assay. A greater number of cells was observed in the negative control group (Fig. 4B), C-JDBM (Fig. 4F) and TC4 (Fig. 4H) groups after 3 days, as compared with the other groups (Fig. 4I–L), due to the loss of cells in the center of the other culture plates, while no other differences were observed between them. As shown in Fig. 4A–C and E–G, the number of cells was directly associated with the incubation time. The chondrocytes in the positive control group were extremely scarce (Fig. 4D and P), and unlike the elongated, polygonal, deltoid or irregular shape of the other chondrocytes (Fig. 4M–O and Q–T), their morphology had changed to a small and round shape (Fig. 4P), whereas the cells from all the other treatment groups appeared normal.

### Apoptosis assay

**Figure 5:**
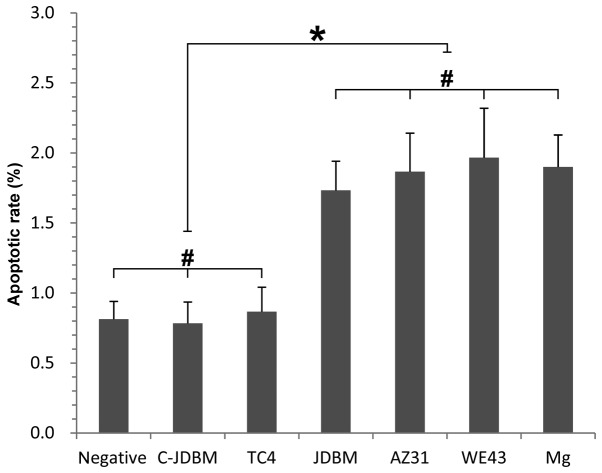
Apoptotic rate of chondrocytes cultured in 25 cm^2^ plates after 5 days of incubation with Mg-Nd-Zn-Zr (JDBM), brushite (CaHPO_4_·_2_H_2_O)-coated JDBM (C-JDBM), AZ31, WE43, pure magnesium (Mg), and TC4 extraction media, as well as in Dulbecco’s modified Eagle’s medium with F12 (DMEM/F12). ^*^P<0.05 (DMEM/F12, C-JDBM and TC4 vs. JDBM, AZ31, WE43, and Mg). ^#^P>0.05 (DMEM/F12 vs. C-JDBM vs. TC4, JDBM vs. AZ31 vs. WE43 vs. Mg). Negative control, cells cultured in DMEM/F12 alone; positive control, cells cultured in DMEM/F12 medium with 10% DMSO.

The results from cell apoptosis assay (Fig. 5) were similar to those obtained by MTT assay and the analysis of cell morphology (Figs. 3 and 4). The cells cultured on C-JDBM and TC4 were comparable with those of the negative control group (p>0.05), in terms of the apoptotic rate, whereas the apoptotic rate of the cells in the other groups was higher (p<0.05). There were no statistically significant differences observed among the JDBM, AZ31, WE43 and pure Mg groups (p> 0. 05).

### Total GAG quantification assay

**Figure 6:**
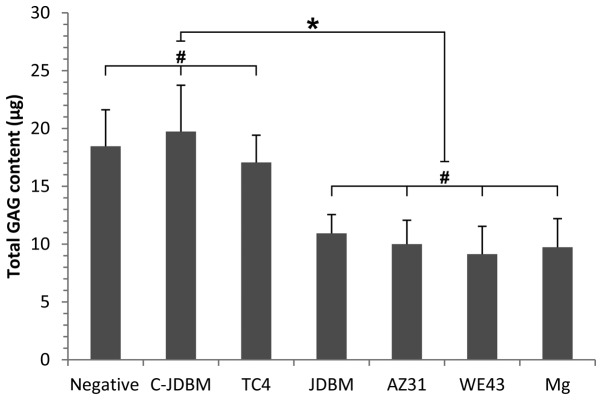
Total glycosaminoglycan (GAG) content of chondrocytes cultured in 25 cm^2^ plates after 5 days of incubation with Mg-Nd-Zn-Zr (JDBM), brushite (CaHPO_4_·_2_H_2_O)-coated JDBM (C-JDBM), AZ31, WE43, pure magnesium (Mg), and TC4 extraction mediums, as well as Dulbecco’s modified Eagle’s medium with F12 (DMEM/F12). ^*^P<0.05 (DMEM/F12, C-JDBM and TC4 vs. JDBM, AZ31, WE43, and Mg). ^#^P>0.05 (DMEM/F12 vs. C-JDBM vs. TC4, JDBM vs. AZ31 vs. WE43 vs. Mg). Negative control, cells cultured in DMEM/F12 alone; positive control, cells cultured in DMEM/F12 medium with 10% DMSO.

The results obtained from the analysis of the GAG content (Fig. 6) were comparable to those obtained by MTT assay and the analysis of cell morphology (Figs. 3 and 4) and apoptosis assay (Fig. 5). The GAG content in the cells cultured on C-JDBM and TC4 was comparable to that of the negative control, while the GAG content did not differ between the cells in the other groups. There was no statistically significant difference observed in the GAG content between the JDBM, AZ31, WE43 and pure Mg groups (p>0.05).

### Col II content and ELISA

The results from ELISA were not satisfactory as the values obtained for the JDBM, AZ31, WE43 and Mg samples were extremely low, much lower than those of the kit (0.1–30 ng/ml). Moreover, the negative control, as well as the C-JDBM and TC4 groups, only had values of 0.112±0.0083, 0.122±0.0068 and 0.101±0.0089 ng/ml, respectively (data not shown, as these results were lower than the lower limit of the measuring range of the kit).

### RT-qPCR

**Figure 7:**
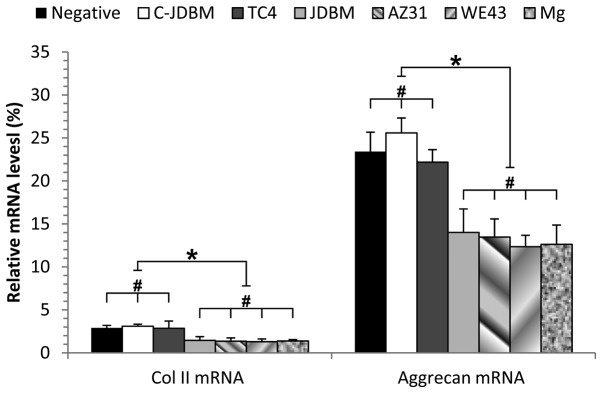
Aggrecan and collagen II (Col II) gene expression relative to GAPDH after 5 days of incubation with the Mg-Nd-Zn-Zr (JDBM), brushite (CaHPO_4_·_2_H_2_O)-coated JDBM (C-JDBM), AZ31, WE43, pure magnesium (Mg), and TC4 extraction media, with Dulbecco’s modified Eagle’s medium with F12 (DMEM/F12) as the control. *P<0.05 (DMEM/F12, C-JDBM and TC4 vs. JDBM, AZ31, WE43, and Mg). ^#^P>0.05 (DMEM/F12 vs. C-JDBM vs. TC4, JDBM vs. AZ31 vs. WE43 vs. Mg). Negative control, cells cultured in DMEM/F12 alone; positive control, cells cultured in DMEM/F12 medium with 10% DMSO.

As shown in Fig. 7, the results of the analysis of the relative mRNA expression levels of aggrecan and Col II in the chondrocytes following 5 days of incubation under the different treatment conditions were consistent with those obtained form the other assays (Figs. 3–6) and with the results of our previous study (12). The results revealed that the cytocompatibility of JDBM was comparable to that of AZ31, WE43 and pure Mg. Furthermore, the CaHPO_4_·_2_H_2_O coating significantly improved its compatibility.

## Discussion

Despite numerous studies on Mg alloys for biomedical applications, the majority of these studies focused on the commercial Mg alloys that may be harmful to the human body (8–10). The JDBM alloy was originally developed as a Mg alloy for medical implants (11,12,15). The main aim of this study was to evaluate the cytocompatibility of JDBM to chondrocytes, which may be used for cartilage tissue engineering. The biocompatibility of different cell lines with the same Mg alloy may be significantly different (16). However, chondrocytes, widely used as ‘seed’ cells in cartilage tissue engineering, have been rarely used to study the biocompatibility of Mg alloys. Our preliminary study presented positive results (12); thus, further investigation was performed. In the present study, JDBM and C-JDBM served as the test groups, whereas the AZ31, WE43, pure Mg and TC4 alloy group were set up as the control group, aside from the negative and positive controls. Comparisons among groups revealed that JDBM and C-JDBM had better reference values as regards their cytocompatibility, based on previous positive results obtained for AZ31, WE43, and TC4 alloys, as well as pure Mg (1,2,17–19).

Cell direct adhesion experiments revealed efficient chondrocyte growth, distribution and adhesion to all material surfaces (Figs. 1 and 2). The adhesion behavior of the chondrocytes is similar to that of MG-63 cells, L929 cells, human bone marrow stromal cells (hBMSCs) and MC3T3-E1 cells on the surface of Mg alloys (7,17,20,21). Furthermore, as shown in Fig. 2, the number of cells that had adhered to the surface of the C-JDBM and TC4 discs (Figs. 2A and F) was greater than that of the other groups (Fig. 2B–E), thereby indicating the better biocompatibility of chondrocytes with C-JDBM and TC4 than the others. Although the cells cultured on the WE43 alloy appeared to be fewer (Fig. 2D), the biocompatibility of WE43 is not necessarily worse than that of other non-coating materials. According to the study by Witte *et al* (22), the direct cell assay reduces cell viability more rapidly than the indirect cytotoxicity tests. Cells are known to be very sensitive to environmental fluctuations, including ion release, changes in pH and hydrogen evolution. The disintegrated particles and corrosion product Mg(OH)_2_ for Mg-based biomaterials, as well as the influencing factors increased when the cells were directly exposed to the material. To be specific, the significant increase in the pH of cell culture media caused by Mg alloy degradation may have an adverse effect on the cells (8,18,22-24). Besides, a corrosion product layer is formed during the corrosion process of Mg and Mg alloys. During immersion, the corrosion product gradually falls from the surface due to the severe mismatch between the substrate and the corrosion product layer (1). The formed corrosion product and departure process make it difficult for the cells to attach to the surface. Furthermore, the high hydrogen evolution rate affects cell attachment and proliferation. Once a Mg-based material is immersed in the cell culture medium, the hydrogen gas evolves from the sample surface, which may deteriorate cell adhesion and the ensuing proliferation process. With a high pH value, Mg(OH)_2_ and zinc hydroxide [Zn(OH)_2_] precipitate easily during the incubation period from the surface of the sample, but also in the culture medium. However, the toxicity of micro-sized Zn(OH)_2_ may be related to the particle concentration. Nair *et al* (25) indicated that micro-ZnO is extremely toxic to MG63 cells at a concentration above 100 *µ*m (with an ~50% loss in cell viability). There were different environment fluctuations for each sample. Due to the aforementioned reasons, we changed the medium once a day to reduce the influence of these fluctuations on the cells.

The SEM micrograph revealed few chondrocytes on the TC4 disc. However, a few cells were fluorescently stained on the parallel sample (Fig. 2F). The lower adhesive force of chondrocytes, as compared with other cells may account for this result. Chondrocyte digestion possibly occurs more rapidly than other cells, including MC3T3-E1 cells in flask cultures; it only needs approximately 30–60 sec. Thus, the chondrocytes may have easily dropped off from the surface of the TC4 samples due to their complex treatment process prior to SEM. The shadows in Fig. 1F show the chondrocytes spreading and falling off. Moreover, Mg alloys have rougher surfaces following degradation than the Ti alloy, which then favors chondrocyte adhesion onto Mg alloys.

Based on indirect cell cytotoxicity and proliferation assay, the cytocompatibility of JDBM was comparable with that of AZ31, WE43 and pure Mg; however, it may be significantly improved by CaHPO_4_·_2_H_2_O coating. Cell viability increased with time, which was in accordance with the results obtained by direct and indirect adhesion assays (Figs. 2 and 4), as well as those of previous studies (7,16,17,20). The cell counts in parallel 96-well plates were consistent with those obtained by MTT assay and the anlaysis of cell morphology (Figs. 3 and 4). A greater number of cells was observed in the negative control, C-JDBM and TC4 groups. Some cells had disappeared from the center of the plates in the JDBM, AZ31, WE43 and pure Mg groups. The following reasons may account for these results: firstly, the obvious increase in the pH in the media caused by Mg alloy corrosion had an adverse effect on cell viability, as previously mentioned (7,8,17,19,23,24). Our preliminary results presented high pH values for uncoated samples (12); however, further and more detailed investigations are warranted to clarify this issue. Secondly, chondrocytes can be expanded *in vitro* in monolayer culture, although this multiplication may lead to dedifferentiation beginning from 3 or 4 passages. This process makes the cells become fibroblast-like as they lose their round phenotype and become spindle-shaped, while switching their collagen production from types II, IX and XI to types I, III and V (26–29). Given the limitations of a low initial number of cells and their dedifferentiation, chondrocytes are multiplied in monolayer culture to increase the number of cells and are then transferred to a three-dimensional culture system to regain their phenotype (26,27,29). Thus, in the present study, we used cells that were passaged twice to eliminate such limitations. Apparently, the viability of the chondrocytes is inferior to that of other cells (26,27,29). Thirdly, the wells of 96-well plates are small, and surface tension brings more cell suspensions to the periphery, and more cells gathered together are more susceptible to survival than sparse ones.

The results obtained by MTT and apoptosis assays, as well as the from the analysis of GAG and Col II content alongside the relative mRNA expression of aggrecan and Col II were likewise similar. GAG, Col II, aggrecan and Col II mRNA expression are unique to chondrocytes (14,26–29). The cells were visibly well attached to the coating and proliferated normally. These results can be explained by the corrosion protecting effect of the brushite coating, as discussed above, thereby improving the cytocompatibility. The purity of Mg alloys and surface modification may reduce the degradation rate of Mg and its alloys (7,17,19,20,22,30). Mg purity has improved as metallurgical techniques have improved. Surface modification includes alkali-heat treatment, carbonate treatment, ion plating deposition of Ti, surface plasma immersion ion implantation, micro-arc oxidation, fluoride coating and phosphate coating. Calcium phosphate (Ca-P) coatings are widely used on bone implant materials due to their favorable biocompatibility and osteoconductive properties (7,31). The brushite (CaHPO_4_·_2_H_2_O) coating has been reported to significantly improve the biocorrosion resistance and osseous integration of Mg alloys (7). In this study, CaHPO_4_·_2_H_2_O was coated onto JDBM alloys through chemical deposition. Compared with other surface treatment methods, the CaHPO_4_·_2_H_2_O coating is simpler and easier to control. Furthermore, CaHPO_4_·_2_H_2_O is applicable to implants of complex shapes and low-temperature processes. This last feature is of particular importance during the surface modification of Mg implants due to the low melting point of Mg (17,30). The Ca-P coating can effectively reduce the degradation rate of Mg and its alloys. Our findings confirm the excellent biocompatibility and desirable protective effects of the coated sample, which is in agreement with the results previously reported (7,17,20,30,31).

The property difference of our proposed coated and uncoated alloys may be attributed to the following reasons: firstly, the alkalization effect caused by the rapid corrosion of Mg alloys is undesirable for cell adhesion, growth and proliferation; this alkalization effect rapidly increases the pH of the SBF until pH >9.0 within approximately 2 h, which is beyond the pH range suitable for cell survival, before it finally reaches a relatively stable value of 10.5 (32). According to previously reported results, the Ca-P coating can serve as an effective corrosion inhabiting layer and reduce the increase in pH of simulated body fluid (33). Although cells are very sensitive to environmental fluctuations, particularly fluctuations in pH (22–24), a Ca-P coating can improve the corrosion resistance of JDBM alloys and provide an environment for cells with a suitable pH (12,30). Thus, the Ca-P coating would likewise benefit cell adhesion and growth. The *in vitro* cell assays, including cell adhesion, MTT, apoptosis, GAG content assay and RT-PCR, demonstrated that C-JDBM had an excellent cellular response due to the CaHPO_4_·_2_H_2_O coating.

Mg and Ca ions have been previouly demonstrated to promote cell viability and proliferation, which are known to promote cell differentiation (7,34). Mg ions at an appropriate concentration can activate bone cells by influencing the protein synthesis and ancillary processes (2,34). Furthermore, Mg ion concentrations of up to 10.286 mM have been reported to be safe to human bone marrow-derived stromal cells (24). Another study demonstrated that physiologically high extracellular Mg concentrations (10 mM) enhance chondrocyte proliferation and redifferentiation in distinct concentration ranges (16). Mg is likewise known to be active in cell adhesion mechanisms (35). Furthermore, Mg hydroxide may enhance osteoblast activity and decrease the osteoclast number temporarily in peri-implant bone remodeling (36). Overall, the Mg ion and Mg(OH)_2_ may enhance the viability, proliferation, and adhesion ability of chondrocytes.

Finally, calcium (Ca) ions are essential in chemical signaling for cells (37). Ca ions on the material surfaces favor protein absorption, e.g., fibronectin and vitronectin, which are important cell attachment-promoting proteins. Therefore, Ca ions improve cell attachment and spreading onto the surface (38). The enhanced Mg ion concentration elevates the calcium concentration (39), which is suitable for the nucleation of the Ca-P containing compounds. This mechanism verifies that Mg is capable of osteoconductivity (39,40). Bone growth on an implant surface requires the presence of sufficient amounts of Ca and phosphate ions (17). Therefore, CaHPO_4_·_2_H_2_O enhances the cellular response.

The combined results show that the presence of Ca and Mg ions, together with a more stable pH, may increase cell proliferation with C-JDBM, as compared with the uncoated JDBM, AZ31, WE43 and Ti alloys or pure Mg (12).

In conclusion, in this study, the Mg alloy, JDBM, was investigated as a medical biodegradable material in terms of its cytocompatibility to chondrocytes *in vitro*. JDBM demonstrated high biocompatibility to chondrocytes, which was similar to the performance of AZ31, WE43 and pure Mg. The CaHPO_4_·_2_H_2_O coating may significantly improve its biocompatibility, which was attributed to the presence of Mg and Ca ions, as well as its more stable pH.

## Figures and Tables

**Table I:** Statistical analysis of time, group factor effect and day x group interaction effect.

Mauchly’s test of sphericity^a^								

Within subjects effect	Mauchly’s W		Approx. Chi-square	Df	Sig.	ε^b^		
						Greenhouse-Geisser	Huynh-Feldt	Lower-bound

Day	0.917		3.380	2	0.185	0.923	1.000	0.500
Tests of within subjects effects								

Source			Type III sum of squares		Df	Mean square	F-value	Sig.

Day								
Sphericity assumption			1.597		2	0.798	475.791	0.000
Greenhouse-Geisser			1.597		1.847	0.865	475.791	0.000
Huynh-Feldt			1.597		2.000	0.798	475.791	0.000
Lower-bound			1.597		1.000	1.597	475.791	0.000
Day x group								
Sphericity assumption			0.802		14	0.057	34.132	0.000
Greenhouse-Geisser			0.802		12.927	0.062	34.132	0.000
Huynh-Feldt			0.802		14.000	0.057	34.132	0.000
Lower-bound			0.802		7.000	0.115	34.132	0.000
Error (day)								
Sphericity assumption			0.134		80	0.002		
Greenhouse-Geisser			0.134		73.868	0.002		
Huynh-Feldt			0.134		80.000	0.002		
Lower-bound			0.134		40.000	0.003		

Tests of between subjects effects								

Source	Type III sum of squares			Df		Mean square	F-value	Sig.

Intercept	15.060			1		15.060	3.608E3	0.000
Group	4.437			7		0.634	151.828	0.000
Error	0.167			40		0.004		

Mauchly’s W, value of Mauchly’s W statistic; Approx. Chi-square, approximate Chi-square statistical value; Df, degrees of freedom of the Chi-square statistic; Sig., significance.

a Use of ‘Mauchly’s test of sphericity’;

b As the value was P>0.185, it need to be corrected, and we used ‘Mauchly’s W, and thus we did not need to correct the ε value ‘Greenhouse Geisser, Huynh Feldt, Lower bound’.

## References

[1] Song G, Atrens A, St John D, Wu X, Nairn J (1997). The anodic dissolution of magnesium in chloride and sulphate solutions. Corros Sci.

[2] Witte F, Kaese V, Haferkamp H, Switzer E, Meyer-Lindenberg A, Wirth CJ, Windhagen H (2005). In vivo corrosion of four magnesium alloys and the associated bone response. Biomaterials.

[3] Witte F, Fischer J, Nellesen J, Crostack HA, Kaese V, Pisch A, Beckmann F, Windhagen H (2006). In vitro and in vivo corrosion measurements of magnesium alloys. Biomaterials.

[4] Xu L, Yu G, Zhang E, Pan F, Yang K (2007). In vivo corrosion behavior of Mg-Mn-Zn alloy for bone implant application. J Biomed Mater Res A.

[5] Kannan MB, Raman RKS (2008). In vitro degradation and mechanical integrity of calcium-containing magnesium alloys in modified-simulated body fluid. Biomaterials.

[6] Staiger MP, Pietak AM, Huadmai J, Dias G (2006). Magnesium and its alloys as orthopedic biomaterials: A review. Biomaterials.

[7] Xu L, Pan F, Yu G, Yang L, Zhang E, Yang K (2009). In vitro and in vivo evaluation of the surface bioactivity of a calcium phosphate coated magnesium alloy. Biomaterials.

[8] Witte F, Hort N, Vogt C, Cohen S, Kainer KU, Willumeit R, Feyerabend F (2008). Degradable biomaterials based on magnesium corrosion. Curr Opin Solid State Mater Sci.

[9] El-Rahman SS (2003). Neuropathology of aluminum toxicity in rats (glutamate and GABA impairment). Pharmacol Res.

[10] Hirano S, Suzuki KT (1996). Exposure, metabolism, and toxicity of rare earths and related compounds. Environ Health Perspect.

[11] Yuan G, Zhang X, Niu J, Tao H, Chen D, He Y, Jiang Y, Ding W (2011). Research progress of new type of degradable biomedical magnesium alloys JDBM. Chin J Nonferrous Met.

[12] Liao Y, Ouyang Y, Niu J, Zhang J, Wang Y, Zhu Z, Yuan G, He Y, Jiang Y (2012). In vitro response of chondrocytes to a biodegradable Mg-Nd-Zn-Zr alloy. Mater Lett.

[13] Niu J, Yuan G, Liao Y, Mao L, Zhang J, Wang Y, Huang F, Jiang Y, He Y, Ding W (2013). Enhanced biocorrosion resistance and biocompatibility of degradable Mg-Nd-Zn-Zr alloy by brushite coating. Mater Sci Eng C Mater Biol Appl.

[14] Lin YJ, Yen CN, Hu YC, Wu YC, Liao CJ, Chu IM (2009). Chondrocytes culture in three-dimensional porous alginate scaffolds enhanced cell proliferation, matrix synthesis and gene expression. J Biomed Mater Res A.

[15] Zhang XB, Yuan GY, Mao L, Niu JL, Ding WJ (2012). Biocorrosion properties of as-extruded Mg-Nd-Zn-Zr alloy compared with commercial AZ31 and WE43 alloys. Mater Lett.

[16] Feyerabend F, Fischer J, Holtz J, Witte F, Willumeit R, Drücker H, Vogt C, Hort N (2010). Evaluation of short-term effects of rare earth and other elements used in magnesium alloys on primary cells and cell lines. Acta Biomater.

[17] Geng F, Tan LL, Jin XX, Yang JY, Yang K (2009). The preparation, cytocompatibility, and in vitro biodegradation study of pure β-TCP on magnesium. J Mater Sci Mater Med.

[18] Popat KC, Leoni L, Grimes CA, Desai TA (2007). Influence of engineered titania nanotubular surfaces on bone cells. Biomaterials.

[19] Keim S, Brunner JG, Fabry B, Virtanen S (2011). Control of magnesium corrosion and biocompatibility with biomimetic coatings. J Biomed Mater Res B Appl Biomater.

[20] Li J, Song Y, Zhang S, Zhao C, Zhang F, Zhang X, Cao L, Fan Q, Tang T (2010). In vitro responses of human bone marrow stromal cells to a fluoridated hydroxyapatite coated biodegradable Mg-Zn alloy. Biomaterials.

[21] Zhang SX, Li JA, Song Y, Zhao C, Zhang X, Xie C, Zhang Y, Tao H, He Y, Jiang Y, Bian YJ (2009). In vitro degradation, hemolysis and MC3T3-E1 cell adhesion of biodegradable Mg-Zn alloy. Mater Sci Eng C Mater Biol Appl.

[22] Witte F, Feyerabend F, Maier P, Fischer J, Störmer M, Blawert C, Dietzel W, Hort N (2007). Biodegradable magnesium-hydroxy-apatite metal matrix composites. Biomaterials.

[23] Serre CM, Papillard M, Chavassieux P, Voegel JC, Boivin G (1998). Influence of magnesium substitution on a collagen-apatite biomaterial on the production of a calcifying matrix by human osteoblasts. J Biomed Mater Res.

[24] Yang C, Yuan G, Zhang J, Tang Z, Zhang X, Dai K (2010). Effects of magnesium alloys extracts on adult human bone marrow-derived stromal cell viability and osteogenic differentiation. Biomed Mater.

[25] Nair S, Sasidharan A, Divya Rani VV, Menon D, Nair S, Manzoor K, Raina S (2009). Role of size scale of ZnO nanoparticles and microparticles on toxicity toward bacteria and osteoblast cancer cells. J Mater Sci Mater Med.

[26] Chaipinyo K, Oakes BW, van Damme MPI (2002). Effects of growth factors on cell proliferation and matrix synthesis of low-density, primary bovine chondrocytes cultured in collagen I gels. J Orthop Res.

[27] Brodkin KR, García AJ, Levenston ME (2004). Chondrocyte phenotypes on different extracellular matrix monolayers. Biomaterials.

[28] Martin I, Suetterlin R, Baschong W, Heberer M, Vunjak-Novakovic G, Freed LE (2001). Enhanced cartilage tissue engineering by sequential exposure of chondrocytes to FGF-2 during 2D expansion and BMP-2 during 3D cultivation. J Cell Biochem.

[29] Gagne TA, Chappell-Afonso K, Johnson JL, McPherson JM, Oldham CA, Tubo RA, Vaccaro C, Vasios GW (2000). Enhanced proliferation and differentiation of human articular chondrocytes when seeded at low cell densities in alginate in vitro. J Orthop Res.

[30] Du H, Wei Z, Wang H, Zhang E, Zuo L, Du L (2011). Surface microstructure and cell compatibility of calcium silicate and calcium phosphate composite coatings on Mg-Zn-Mn-Ca alloys for biomedical application. Colloids Surf B Biointerfaces.

[31] Hench LL (1998). Bioceramics. J Am Ceram Soc.

[32] Song GL, Song SZ (2007). A possible biodegradable magnesium implant material. Adv Eng Mater.

[33] Xu L, Zhang E, Yang K (2009). Phosphating treatment and corrosion properties of Mg-Mn-Zn alloy for biomedical application. J Mater Sci Mater Med.

[34] Rude RK, Gruber HE, Wei LY, Frausto A, Mills BG (2003). Magnesium deficiency: Effect on bone and mineral metabolism in the mouse. Calcif Tissue Int.

[35] Paul W, Sharma CP (2006). Nanoceramic matrices: Biomedical applications. Am J Biochem Biotechnol.

[36] Janning C, Willbold E, Vogt C, Nellesen J, Meyer-Lindenberg A, Windhagen H, Thorey F, Witte F (2010). Magnesium hydroxide temporarily enhancing osteoblast activity and decreasing the osteoclast number in peri-implant bone remodelling. Acta Biomater.

[37] Ilich JZ, Kerstetter JE (2000). Nutrition in bone health revisited: A story beyond calcium. J Am Coll Nutr.

[38] Feng B, Weng J, Yang BC, Qu SX, Zhang XD (2004). Characterization of titanium surfaces with calcium and phosphate and osteoblast adhesion. Biomaterials.

[39] TenHuisen KS, Brown PW (1997). Effects of magnesium on the formation of calcium-deficient hydroxyapatite from CaHPO _4_·2H_2_O and Ca_4_(PO_4_)_2_O. J Biomed Mater Res.

[40] Witte F, Reifenrath J, Müller PP, Crostack H-A, Nellesen J, Bach FW, Bormann D, Rudert M (2006). Cartilage repair on magnesium scaffolds used as a subchondral bone replacement. Materialwiss Werkstofftech.

